# IMMUNOLOGICAL ASPECTS OF AGING

**DOI:** 10.1093/geroni/igae098.1285

**Published:** 2024-12-31

**Authors:** Matt Yousefzadeh

**Affiliations:** Chair

## Abstract

Aging is an intricate and multifaceted phenomenon that impacts every aspect of our bodies, including the immune system. As we age, there’s a gradual decline in immune function known as immunosenescence, which reduces the body’s ability to combat pathogens effectively. Moreover, this decline in immune function plays a pivotal role in the development of various age-related chronic conditions. One significant contributor to aging is inflammaging or the production of low-grade chronic inflammation that occurs in the absence of pathogens that can drive aging. Despite significant research efforts, the intricate mechanisms behind immunosenescence and the communication of aging between the immune system and other bodily systems remain largely elusive. The aim of the symposium is to delve into recent breakthroughs in this field across different levels of research, spanning from basic science, translational studies, and potential therapeutic interventions.

